# Crystal structure of a 2:1 piroxicam–gentisic acid co-crystal featuring neutral and zwitterionic piroxicam mol­ecules

**DOI:** 10.1107/S2056989016017321

**Published:** 2016-11-04

**Authors:** Elizabeth M. Horstman, Jeffery A. Bertke, Toby J. Woods, Paul J. A. Kenis

**Affiliations:** a University of Illinois, Department of Chemical and Biomolecular Engineering, 600 S. Mathews Ave, Urbana, IL 61801, USA; b University of Illinois, School of Chemical Sciences, Box 59-1, 505 South Mathews Avenue, Urbana, Illinois 61801, USA

**Keywords:** crystal structure, hydrogen bonding, piroxicam

## Abstract

A new 2:1 co-crystal of piroxicam and gentisic acid [systematic name: 4-hy­droxy-1,1-dioxo-*N*-(pyridin-2-yl)-2*H*-1λ^6^,2-benzo­thia­zine-3-carboxamide–2-(4-oxido-1,1-dioxo-2*H*-1λ^6^,2-benzo­thia­zine-3-amido)­pyridin-1-ium–2,5-di­hydroxy­benzoic acid, 2C_15_H_13_N_3_O_4_S·C_7_H_6_O_4_] has been synthesized using a microfluidic platform and initially identified using Raman spectroscopy. In the co-crystal, one piroxicam mol­ecule is in its neutral form and an intra­molecular O—H⋯O hydrogen bond is observed. The other piroxicam mol­ecule is zwitterionic (proton transfer from the OH group to the pyridine N atom) and two intra­molecular N—H⋯O hydrogen bonds occur. The gentisic acid mol­ecule shows whole-mol­ecule disorder over two sets of sites in a 0.809 (2):0.191 (2) ratio. In the crystal, extensive hydrogen bonding between the components forms layers propagating in the *ab* plane.

## Abstract 2

A new co-crystal of piroxicam and gentisic acid has been characterized, in which the ratio of piroxicam (one neutral mol­ecule and one zwitterion) to gentisic acid is 2:1.

## Chemical context   

Piroxicam is a non-steroidal anti-inflammatory drug classified as a BCS Class II drug due to its low aqueous solubility (Amidon *et al.*, 1995 ▸; Thayer, 2010 ▸). Co-crystallization of an active pharmaceutical ingredient (API) and an FDA-approved counter-ion is a common technique employed to increase the solubility of the API (Trask *et al.*, 2005 ▸). In this work, we explored the co-crystallization of piroxicam and gentisic acid. In a previous study, piroxicam was co-crystallized with 23 carb­oxy­lic acids yielding 50 co-crystals. From this work, three co-crystals of piroxicam and gentisic acid were identified with Raman spectroscopy, but no crystal structures were reported (Childs & Hardcastle, 2007 ▸). In our prior work, we reported the crystal structure of two co-crystals of piroxicam and gentisic acid, one was a 1:1 co-crystal and the second was a solvated co-crystal that incorporated acetone into the crystal in a 1:1:1 molar ratio (Horstman *et al.*, 2015 ▸). In this work we describe the crystal structure of a 2:1 piroxicam:gentisic acid co-crystal.

## Structural commentary   

**Figure 1:**
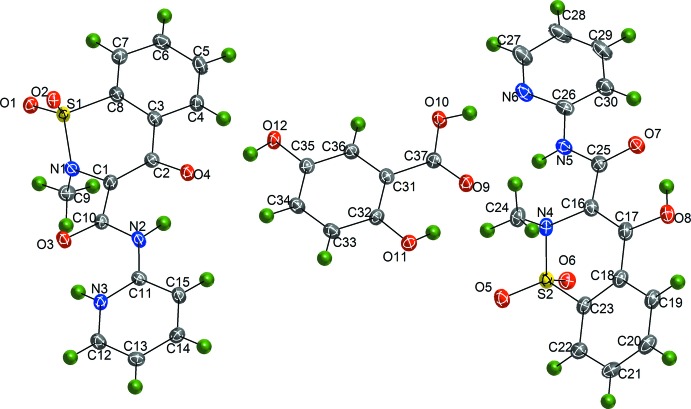
The mol­ecular structure of the title co-crystal, showing 35% probability ellipsoids for non-H atoms and spheres of arbitrary size for H atoms. Only the major component of the disordered gentisic acid is shown.

The asymmetric unit of this co-crystal consists of two piroxicam mol­ecules and one gentisic acid mol­ecule, with all atoms residing on general positions (Fig. 1 ▸). One of the piroxicam mol­ecules is neutral and the other is a zwitterion: the two mol­ecules exhibit two different conformations in the crystal structure. In the neutral S2-containing mol­ecule, intra­molecular hydrogen bonding exists between the hydroxyl proton H8 [H⋯*A* = 1.79 (3) Å] and the amide oxygen atom O7. In the S2 mol­ecule, free rotation about the C—C bond (C1 and C10) allows a second zwitterionic conformation in which intra­molecular hydrogen bonds exist between the amine proton H2 and the enolate oxygen atom O4 [H⋯*A* = 1.85 (2) Å] and the pyridinium proton H3 and the amide oxygen atom O3 [H⋯*A* = 2.19 (2) Å]. Further details of the hydrogen bonding are provided in Table 1 ▸.
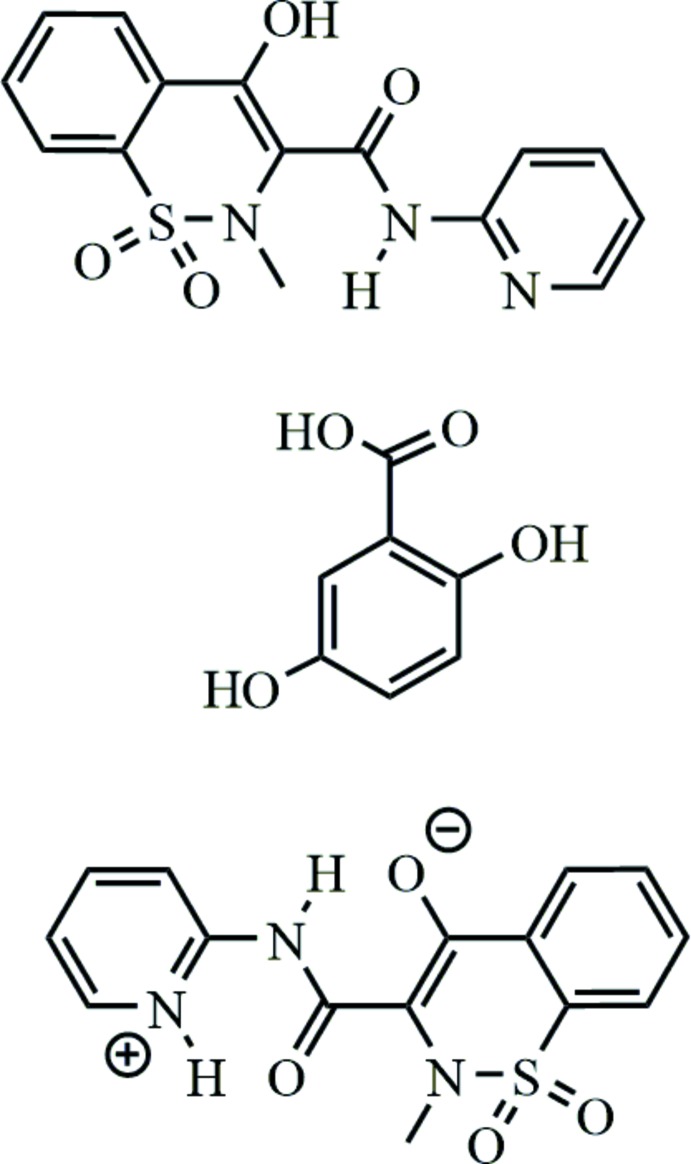


The gentisic acid mol­ecule shows whole mol­ecule disorder over two orientations rotated by approximately 180° in the plane of the aromatic ring with site occupancies of 0.809 (2):0.191 (2). The major orientation participates in intra­molecular hydrogen bonding between the O11 hydroxide substituent of the benzene ring and the O9 oxygen atom of the carb­oxy­lic acid [H⋯*A* = 1.89 (3) Å]. The major orientation also participates in inter­molecular hydrogen bonding. The minor orientation of the gentisic acid mol­ecule also displays intra­molecular hydrogen bonding between the O11*B* hydroxide substituent and the O9*B* oxygen atom of the carb­oxy­lic acid (H⋯*A* = 1.89 Å) but does not participate in inter­molecular hydrogen bonding.

## Supra­molecular features   

**Figure 2:**
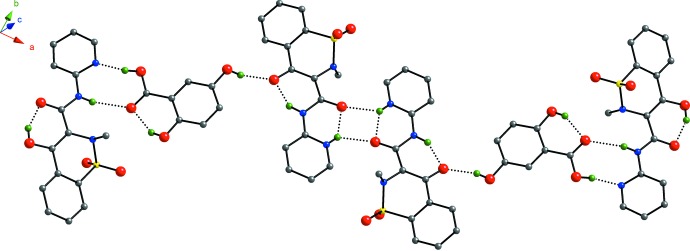
Ball-and-stick model highlighting the hydrogen-bonding network in the title co-crystal. Only the major component of the disordered gentisic acid is shown. Color key: C gray, N blue, H green, O red, and S yellow. H atoms not involved in hydrogen bonding have been omitted for clarity.

**Figure 3:**
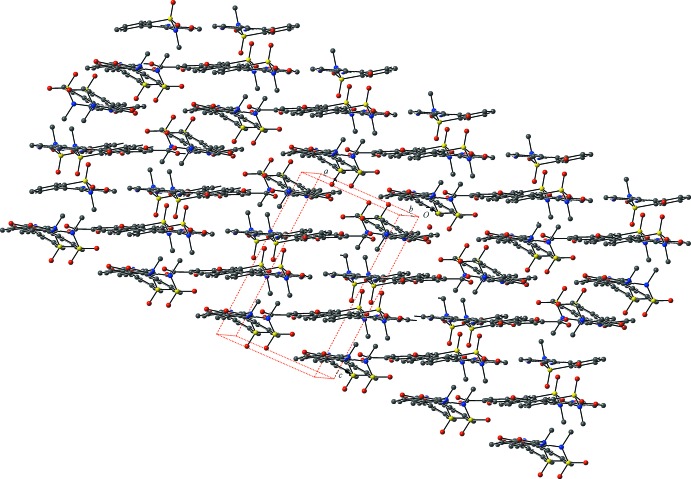
Ball-and-stick packing diagram of the co-crystal, as viewed approximately down the *b* axis, highlighting the layers formed by the packing of the hexa­meric units. Color key: C gray, N blue, O red, and S yellow. H atoms have been omitted for clarity.

In the crystal, hydrogen bonds (Table 1 ▸) between the piroxicam and gentisic acid mol­ecules form hexa­meric units that propagate along the *a-*axis direction (Fig. 2 ▸). These units pack into layers in the *ab* plane; the layers stack along the *c-*axis direction, Fig. 3 ▸.

The repeating motif of the hexa­meric unit is formed by one gentisic acid mol­ecule hydrogen bonded to two piroxicam mol­ecules, one of each conformation (*i.e*. neutral and zwitterion). The non-zwitterionic form of piroxicam accepts hydrogen bonds from the gentisic acid *via* O—H⋯N bonds between the carb­oxy­lic acid and the pyridine ring of piroxicam [H⋯*A* = 1.74 (3) Å] as well as N—H⋯O bonds between the carbonyl oxygen atom of gentisic acid and the amine nitro­gen atom of piroxicam [H⋯*A* = 2.25 (2) Å]. The zwitterionic form of piroxicam accepts hydrogen bonds from the gentisic acid *via* O—H⋯O bonds between the 5-hy­droxy substituent of gentisic acid and the enolate oxygen atom of piroxicam [H⋯*A* = 1.94 (3) Å]. The repeat units are linked together by N—H⋯O bonds between the pyridinium nitro­gen atoms and the amide oxygen atoms of the zwitterionic form of piroxicam [H⋯*A* = 2.19 (2) Å].

## Synthesis and crystallization   

Piroxicam (>=98.0%) and gentisic acid (>=98.0%) were used as purchased from Sigma–Aldrich (St. Louis, MO, USA). Aceto­nitrile (>=99.9%) was used as purchased from Fisher Scientific (Fair Lawn, NJ, USA). A 1:2 molar ratio of piroxicam:gentisic acid was dissolved in aceto­nitrile. The concentration of piroxicam in aceto­nitrile was near saturation (∼0.034 *M*). The resulting solution was introduced into a microfluidic platform. The microfluidic platform was a 6 × 6 array of single microwells (∼100 nl) (Horstman *et al.*, 2015 ▸). After being filled, the microfluidic platform was placed inside a petri dish and then the petri dish was sealed with parafilm to slow the rate of solvent evaporation. The crystallization solution evaporated over the course of one day, after which crystals were observed *via* optical microscopy. Specifics of the microfluidic platform fabrication and operation have been previously reported (Horstman *et al.*, 2015 ▸). Once crystals were observed, Raman spectroscopy was used to distinguish between crystals. Within one microfluidic chip, three different co-crystals of piroxicam and gentisic acid were observed, two of which had been previously reported (Horstman *et al.*, 2015 ▸) and one new solid form, reported here. Once the new co-crystals had been identified, we removed the crystals from the microfluidic platform.

## Refinement   

Crystal data, data collection and structure refinement details are summarized in Table 2 ▸. The gentisic acid mol­ecule shows whole mol­ecule disorder over two sets of sites: the like C—O and C—C distances were restrained to be similar (s.u. 0.01 Å). Similar displacement amplitudes (s.u. 0.01) were imposed on disordered sites overlapping by less than the sum of van der Waals radii.

All O—H and N—H hydrogen atoms were located in the difference map except for those on the minor-disordered component of the gentisic acid. The H atoms located in the difference map were allowed to refine the O—H/N—H bond distances. These H atoms refined to good hydrogen-bonding positions (Hamilton & Ibers, 1968 ▸). The hydroxyl H atoms on the minor-disordered component of gentisic acid were optimized by rotation about *R*—O bonds with idealized O—H and *R*—H distances. These H atoms are also in good hydrogen-bonding locations. Methyl H-atom positions, *R*—CH_3_, were optimized by rotation about *R*—C bonds with idealized C—H, *R*⋯H and H⋯H distances. The remaining H atoms were included as riding idealized contributors. Methyl, hydroxyl and amine H atom *U*_iso_ values were assigned as 1.5*U*_eq_ of the carrier atom; remaining H-atom *U*_iso_ were assigned as 1.2 × carrier *U*_eq_.

The (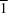 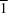
2) reflection was omitted from the final refinement because it was partially obscured by the shadow of the beamstop in some orientations.

## Supplementary Material

Crystal structure: contains datablock(s) I. DOI: 10.1107/S2056989016017321/hb7625sup1.cif

Structure factors: contains datablock(s) I. DOI: 10.1107/S2056989016017321/hb7625Isup2.hkl

Supporting information file. DOI: 10.1107/S2056989016017321/hb7625Isup3.cdx

Supporting information file. DOI: 10.1107/S2056989016017321/hb7625Isup4.cml

CCDC reference: 1512430

Additional supporting information: crystallographic information; 3D view; checkCIF report

## Figures and Tables

**Table 1:** Hydrogen-bond geometry (Å, °)

*D*—H⋯*A*	*D*—H	H⋯*A*	*D*⋯*A*	*D*—H⋯*A*
N2—H2⋯O4	0.88 (2)	1.85 (2)	2.6169 (18)	145.2 (19)
N3—H3⋯O3	0.78 (2)	1.99 (2)	2.6027 (19)	135 (2)
N3—H3⋯O3^i^	0.78 (2)	2.19 (2)	2.8034 (19)	136 (2)
O8—H8⋯O7	0.88 (3)	1.79 (3)	2.5722 (19)	148 (2)
N5—H5⋯O9	0.84 (2)	2.25 (2)	3.074 (2)	167 (2)
O10—H10⋯N6	0.91 (3)	1.74 (3)	2.637 (3)	167 (3)
O11—H11⋯O9	0.81 (3)	1.89 (3)	2.614 (2)	148 (3)
O12—H12⋯O4^ii^	0.81 (3)	1.94 (3)	2.738 (3)	164 (4)
O10*B*—H10*B*⋯O4^ii^	0.84	1.67	2.508 (12)	172
O11*B*—H11*B*⋯O2^i^	0.84	2.57	3.061 (6)	118
O11*B*—H11*B*⋯O9*B*	0.84	1.89	2.614 (9)	143
O12*B*—H12*B*⋯N6	0.84	2.10	2.856 (12)	149

Symmetry codes: (i) 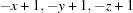; (ii) 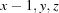.

**Table 2:** Experimental details

Crystal data	
Chemical formula	2C_15_H_13_N_3_O_4_S·C_7_H_6_O_4_
*M*_r_	816.80
Crystal system, space group	Triclinic, *P* 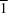
Temperature (K)	105
*a*, *b*, *c* (Å)	8.8764 (3), 13.4060 (5), 15.2678 (6)
α, β, γ (°)	80.4660 (15), 85.4332 (15), 78.4602 (15)
*V* (Å^3^)	1753.48 (11)
*Z*	2
Radiation type	Cu *K*α
μ (mm^−1^)	2.05
Crystal size (mm)	0.15 × 0.15 × 0.03

Data collection	
Diffractometer	Bruker D8 Venture/Photon 100
Absorption correction	Integration (*SADABS*; Bruker, 2014 ▸)
*T*_min_, *T*_max_	0.867, 0.965
No. of measured, independent and observed [*I* > 2σ(*I*)] reflections	49307, 6416, 5801
*R*_int_	0.037
(sin θ/λ)_max_ (Å^−1^)	0.602

Refinement	
*R*[*F* ^2^ > 2σ(*F* ^2^)], *wR*(*F* ^2^), *S*	0.033, 0.086, 1.11
No. of reflections	6416
No. of parameters	637
No. of restraints	374
H-atom treatment	H atoms treated by a mixture of independent and constrained refinement
Δρ_max_, Δρ_min_ (e Å^−3^)	0.24, −0.40

Computer programs: *APEX2*, *SAINT*, *XPREP*, and *XCIF* (Bruker, 2014 ▸), *SHELXS2014-4* and *SHELXTL* (Sheldrick, 2008 ▸), *SHELXL2014-6* (Sheldrick, 2015 ▸) and *CrystalMaker* (CrystalMaker, 1994 ▸).

## supplementary crystallographic information

### Crystal data

**Table:**

2C_15_H_13_N_3_O_4_S·C_7_H_6_O_4_	*Z* = 2
*M**_r_* = 816.80	*F*(000) = 848
Triclinic, *P*1	*D*_x_ = 1.547 Mg m^−^^3^
*a* = 8.8764 (3) Å	Cu *K*α radiation, λ = 1.54178 Å
*b* = 13.4060 (5) Å	Cell parameters from 9375 reflections
*c* = 15.2678 (6) Å	θ = 2.9–68.1°
α = 80.4660 (15)°	µ = 2.05 mm^−^^1^
β = 85.4332 (15)°	*T* = 105 K
γ = 78.4602 (15)°	Plate, colourless
*V* = 1753.48 (11) Å^3^	0.15 × 0.15 × 0.03 mm

### Data collection

**Table:**

Bruker D8 Venture/Photon 100 diffractometer	6416 independent reflections
Radiation source: microfocus sealed tube	5801 reflections with *I* > 2σ(*I*)
Multilayer mirrors monochromator	*R*_int_ = 0.037
profile data from φ and ω scans	θ_max_ = 68.3°, θ_min_ = 2.9°
Absorption correction: integration (SADABS; Bruker, 2014)	*h* = −10→10
*T*_min_ = 0.867, *T*_max_ = 0.965	*k* = −16→15
49307 measured reflections	*l* = −18→18

### Refinement

**Table:**

Refinement on *F*^2^	374 restraints
Least-squares matrix: full	Hydrogen site location: mixed
*R*[*F*^2^ > 2σ(*F*^2^)] = 0.033	H atoms treated by a mixture of independent and constrained refinement
*wR*(*F*^2^) = 0.086	*w* = 1/[σ^2^(*F*_o_^2^) + (0.0348*P*)^2^ + 1.2991*P*] where *P* = (*F*_o_^2^ + 2*F*_c_^2^)/3
*S* = 1.11	(Δ/σ)_max_ = 0.001
6416 reflections	Δρ_max_ = 0.24 e Å^−^^3^
637 parameters	Δρ_min_ = −0.40 e Å^−^^3^

### Special details

**Table:**

Experimental. One distinct cell was identified using APEX2 (Bruker, 2014). Thirty frame series were integrated and filtered for statistical outliers using SAINT (Bruker, 2013) then corrected for absorption by integration using SAINT/SADABS v2014/2 (Bruker, 2014) to sort, merge, and scale the combined data. No decay correction was applied.
Geometry. All esds (except the esd in the dihedral angle between two l.s. planes) are estimated using the full covariance matrix. The cell esds are taken into account individually in the estimation of esds in distances, angles and torsion angles; correlations between esds in cell parameters are only used when they are defined by crystal symmetry. An approximate (isotropic) treatment of cell esds is used for estimating esds involving l.s. planes.
Refinement. Structure was phased by direct methods (Sheldrick, 2014). Systematic conditions suggested the ambiguous space group. The space group choice was confirmed by successful convergence of the full-matrix least-squares refinement on F^2^. The final map had no significant features. A final analysis of variance between observed and calculated structure factors showed little dependence on amplitude and resolution.

### Fractional atomic coordinates and isotropic or equivalent isotropic displacement parameters (Å^2^)

**Table:**

	*x*	*y*	*z*	*U*_iso_*/*U*_eq_	Occ. (<1)
S1	0.90408 (4)	0.14390 (3)	0.54955 (3)	0.01390 (10)	
O1	0.82965 (14)	0.05638 (9)	0.56665 (8)	0.0187 (3)	
O2	0.94543 (13)	0.18072 (9)	0.45902 (8)	0.0171 (3)	
O3	0.63926 (13)	0.43288 (9)	0.53570 (8)	0.0190 (3)	
O4	1.07869 (13)	0.38775 (9)	0.63196 (8)	0.0179 (3)	
N1	0.79315 (16)	0.23889 (11)	0.59094 (9)	0.0146 (3)	
N2	0.82451 (16)	0.51014 (11)	0.57712 (10)	0.0153 (3)	
H2	0.918 (3)	0.4941 (16)	0.5974 (14)	0.023*	
N3	0.60509 (16)	0.63109 (12)	0.53091 (10)	0.0154 (3)	
H3	0.571 (3)	0.5850 (18)	0.5199 (14)	0.023*	
C1	0.86562 (19)	0.32602 (13)	0.59352 (11)	0.0146 (3)	
C2	1.01351 (19)	0.31258 (13)	0.62393 (11)	0.0146 (3)	
C3	1.10283 (19)	0.20511 (13)	0.64669 (11)	0.0145 (3)	
C4	1.22916 (19)	0.18627 (14)	0.70002 (11)	0.0170 (4)	
H4A	1.2584	0.2418	0.7215	0.020*	
C5	1.3123 (2)	0.08704 (14)	0.72188 (12)	0.0208 (4)	
H5A	1.3976	0.0754	0.7586	0.025*	
C6	1.2726 (2)	0.00480 (14)	0.69087 (12)	0.0214 (4)	
H6A	1.3307	−0.0627	0.7062	0.026*	
C7	1.1480 (2)	0.02107 (14)	0.63738 (12)	0.0186 (4)	
H7A	1.1199	−0.0349	0.6158	0.022*	
C8	1.06514 (19)	0.12048 (13)	0.61587 (11)	0.0148 (3)	
C9	0.6952 (2)	0.21086 (14)	0.67045 (12)	0.0203 (4)	
H9A	0.6442	0.1555	0.6607	0.030*	
H9B	0.6174	0.2711	0.6812	0.030*	
H9C	0.7591	0.1874	0.7221	0.030*	
C10	0.76774 (19)	0.42370 (13)	0.56612 (11)	0.0143 (3)	
C11	0.74705 (19)	0.60956 (13)	0.56233 (11)	0.0138 (3)	
C12	0.5198 (2)	0.72695 (13)	0.51690 (11)	0.0176 (4)	
H12A	0.4189	0.7375	0.4961	0.021*	
C13	0.5776 (2)	0.80883 (13)	0.53244 (11)	0.0179 (4)	
H13A	0.5187	0.8768	0.5222	0.022*	
C14	0.7258 (2)	0.79029 (13)	0.56383 (11)	0.0165 (3)	
H14A	0.7689	0.8464	0.5744	0.020*	
C15	0.81005 (19)	0.69159 (13)	0.57964 (11)	0.0159 (3)	
H15A	0.9100	0.6793	0.6021	0.019*	
S2	0.69631 (5)	0.91958 (3)	0.90830 (3)	0.01758 (11)	
O5	0.55243 (14)	0.92712 (10)	0.86939 (9)	0.0249 (3)	
O6	0.71653 (14)	0.87123 (10)	0.99842 (8)	0.0221 (3)	
O7	1.23078 (14)	0.76608 (10)	0.90420 (8)	0.0239 (3)	
O8	1.15980 (15)	0.96135 (11)	0.90637 (8)	0.0224 (3)	
H8	1.218 (3)	0.900 (2)	0.9064 (15)	0.034*	
N4	0.83342 (17)	0.85905 (11)	0.84673 (9)	0.0177 (3)	
N5	1.04254 (18)	0.67834 (12)	0.88379 (10)	0.0197 (3)	
H5	0.950 (3)	0.6828 (18)	0.8715 (15)	0.030*	
N6	1.06042 (17)	0.50686 (12)	0.87478 (11)	0.0243 (4)	
C16	0.9849 (2)	0.86239 (14)	0.87249 (11)	0.0179 (4)	
C17	1.0193 (2)	0.95255 (14)	0.88695 (11)	0.0190 (4)	
C18	0.9022 (2)	1.04731 (14)	0.88701 (11)	0.0186 (4)	
C19	0.9434 (2)	1.14325 (15)	0.88329 (12)	0.0223 (4)	
H19A	1.0489	1.1488	0.8781	0.027*	
C20	0.8303 (2)	1.23013 (15)	0.88712 (13)	0.0251 (4)	
H20A	0.8590	1.2953	0.8839	0.030*	
C21	0.6755 (2)	1.22358 (15)	0.89559 (12)	0.0236 (4)	
H21A	0.5993	1.2840	0.8983	0.028*	
C22	0.6316 (2)	1.12915 (14)	0.90012 (12)	0.0205 (4)	
H22A	0.5259	1.1241	0.9059	0.025*	
C23	0.7451 (2)	1.04244 (14)	0.89609 (11)	0.0177 (4)	
C24	0.8156 (2)	0.88255 (15)	0.74935 (12)	0.0234 (4)	
H24A	0.7116	0.8770	0.7362	0.035*	
H24B	0.8914	0.8335	0.7198	0.035*	
H24C	0.8322	0.9527	0.7276	0.035*	
C25	1.0965 (2)	0.76629 (14)	0.88733 (11)	0.0195 (4)	
C26	1.1243 (2)	0.57710 (14)	0.90290 (12)	0.0190 (4)	
C27	1.1306 (2)	0.40771 (16)	0.89178 (16)	0.0338 (5)	
H27A	1.0865	0.3576	0.8708	0.041*	
C28	1.2628 (2)	0.37473 (16)	0.93788 (16)	0.0339 (5)	
H28A	1.3088	0.3038	0.9490	0.041*	
C29	1.3263 (2)	0.44807 (16)	0.96739 (14)	0.0304 (5)	
H29A	1.4172	0.4278	0.9999	0.036*	
C30	1.2588 (2)	0.55046 (16)	0.94990 (13)	0.0254 (4)	
H30A	1.3025	0.6017	0.9693	0.031*	
O9	0.72760 (18)	0.66660 (12)	0.81992 (11)	0.0214 (4)	0.809 (2)
O10	0.8016 (3)	0.5013 (2)	0.8038 (3)	0.0235 (7)	0.809 (2)
H10	0.888 (4)	0.514 (2)	0.8249 (19)	0.035*	0.809 (2)
O11	0.47005 (19)	0.77049 (14)	0.75304 (11)	0.0209 (4)	0.809 (2)
H11	0.546 (4)	0.758 (2)	0.782 (2)	0.031*	0.809 (2)
O12	0.3599 (3)	0.3988 (2)	0.6873 (2)	0.0234 (6)	0.809 (2)
H12	0.272 (4)	0.407 (3)	0.672 (2)	0.035*	0.809 (2)
C31	0.5536 (2)	0.58575 (16)	0.75879 (14)	0.0152 (5)	0.809 (2)
C32	0.4463 (3)	0.67789 (19)	0.73843 (19)	0.0152 (6)	0.809 (2)
C33	0.3109 (3)	0.67561 (19)	0.69899 (15)	0.0185 (5)	0.809 (2)
H33A	0.2387	0.7377	0.6840	0.022*	0.809 (2)
C34	0.2805 (3)	0.5836 (2)	0.68143 (17)	0.0186 (5)	0.809 (2)
H34A	0.1877	0.5830	0.6546	0.022*	0.809 (2)
C35	0.3854 (3)	0.49164 (17)	0.70295 (14)	0.0172 (5)	0.809 (2)
C36	0.5210 (5)	0.4934 (2)	0.7409 (4)	0.0163 (7)	0.809 (2)
H36A	0.5931	0.4310	0.7551	0.020*	0.809 (2)
C37	0.6998 (3)	0.5882 (2)	0.79668 (14)	0.0167 (5)	0.809 (2)
O9B	0.1664 (7)	0.5901 (5)	0.6578 (4)	0.0206 (16)	0.191 (2)
O10B	0.3138 (14)	0.4318 (6)	0.6853 (10)	0.021 (2)	0.191 (2)
H10B	0.2402	0.4157	0.6631	0.032*	0.191 (2)
O11B	0.2617 (7)	0.7479 (5)	0.6992 (4)	0.0197 (15)	0.191 (2)
H11B	0.2006	0.7193	0.6775	0.030*	0.191 (2)
O12B	0.7796 (13)	0.4710 (8)	0.8181 (12)	0.023 (3)	0.191 (2)
H12B	0.8381	0.5004	0.8411	0.035*	0.191 (2)
C31B	0.4054 (8)	0.5709 (5)	0.7243 (5)	0.0140 (14)	0.191 (2)
C32B	0.3839 (10)	0.6760 (6)	0.7300 (8)	0.011 (2)	0.191 (2)
C33B	0.4961 (9)	0.7113 (8)	0.7689 (7)	0.0185 (18)	0.191 (2)
H33B	0.4817	0.7822	0.7743	0.022*	0.191 (2)
C34B	0.6284 (10)	0.6449 (6)	0.7999 (6)	0.0200 (17)	0.191 (2)
H34B	0.7037	0.6701	0.8263	0.024*	0.191 (2)
C35B	0.6496 (9)	0.5409 (6)	0.7919 (6)	0.0181 (17)	0.191 (2)
C36B	0.5395 (19)	0.5046 (9)	0.7541 (17)	0.016 (3)	0.191 (2)
H36B	0.5550	0.4338	0.7484	0.019*	0.191 (2)
C37B	0.2857 (10)	0.5323 (6)	0.6866 (7)	0.017 (2)	0.191 (2)

### Atomic displacement parameters (Å^2^)

**Table:**

	*U*^11^	*U*^22^	*U*^33^	*U*^12^	*U*^13^	*U*^23^
S1	0.01315 (19)	0.0118 (2)	0.0172 (2)	−0.00249 (15)	−0.00408 (15)	−0.00194 (15)
O1	0.0190 (6)	0.0134 (6)	0.0248 (6)	−0.0051 (5)	−0.0039 (5)	−0.0020 (5)
O2	0.0183 (6)	0.0175 (6)	0.0166 (6)	−0.0050 (5)	−0.0041 (5)	−0.0019 (5)
O3	0.0139 (6)	0.0157 (6)	0.0277 (7)	−0.0021 (5)	−0.0080 (5)	−0.0021 (5)
O4	0.0145 (6)	0.0142 (6)	0.0259 (6)	−0.0031 (5)	−0.0062 (5)	−0.0029 (5)
N1	0.0128 (7)	0.0118 (7)	0.0193 (7)	−0.0025 (5)	−0.0020 (6)	−0.0015 (6)
N2	0.0112 (7)	0.0134 (7)	0.0212 (7)	−0.0022 (6)	−0.0049 (6)	−0.0010 (6)
N3	0.0146 (7)	0.0135 (7)	0.0193 (7)	−0.0040 (6)	−0.0042 (6)	−0.0025 (6)
C1	0.0148 (8)	0.0126 (8)	0.0170 (8)	−0.0037 (7)	−0.0024 (6)	−0.0020 (6)
C2	0.0155 (8)	0.0143 (9)	0.0136 (8)	−0.0025 (7)	−0.0016 (6)	−0.0015 (6)
C3	0.0130 (8)	0.0151 (9)	0.0147 (8)	−0.0021 (7)	−0.0016 (6)	−0.0007 (6)
C4	0.0160 (8)	0.0166 (9)	0.0191 (9)	−0.0042 (7)	−0.0039 (7)	−0.0019 (7)
C5	0.0167 (9)	0.0211 (10)	0.0231 (9)	−0.0019 (7)	−0.0081 (7)	0.0022 (7)
C6	0.0183 (9)	0.0156 (9)	0.0275 (10)	0.0019 (7)	−0.0046 (7)	0.0003 (7)
C7	0.0202 (9)	0.0153 (9)	0.0202 (9)	−0.0028 (7)	−0.0028 (7)	−0.0024 (7)
C8	0.0124 (8)	0.0167 (9)	0.0148 (8)	−0.0019 (7)	−0.0022 (6)	−0.0014 (7)
C9	0.0193 (9)	0.0186 (9)	0.0228 (9)	−0.0055 (7)	0.0025 (7)	−0.0023 (7)
C10	0.0148 (8)	0.0141 (9)	0.0147 (8)	−0.0038 (7)	−0.0013 (6)	−0.0024 (6)
C11	0.0129 (8)	0.0150 (8)	0.0128 (8)	−0.0021 (6)	−0.0002 (6)	−0.0008 (6)
C12	0.0155 (8)	0.0162 (9)	0.0197 (9)	−0.0007 (7)	−0.0047 (7)	−0.0002 (7)
C13	0.0181 (9)	0.0138 (9)	0.0205 (9)	0.0003 (7)	−0.0018 (7)	−0.0024 (7)
C14	0.0183 (8)	0.0158 (9)	0.0162 (8)	−0.0050 (7)	0.0007 (7)	−0.0038 (7)
C15	0.0126 (8)	0.0185 (9)	0.0175 (8)	−0.0041 (7)	−0.0022 (6)	−0.0031 (7)
S2	0.0170 (2)	0.0154 (2)	0.0212 (2)	−0.00564 (16)	−0.00270 (16)	−0.00123 (16)
O5	0.0197 (7)	0.0201 (7)	0.0361 (8)	−0.0052 (5)	−0.0079 (6)	−0.0028 (6)
O6	0.0242 (7)	0.0200 (7)	0.0218 (7)	−0.0076 (5)	0.0007 (5)	0.0004 (5)
O7	0.0175 (6)	0.0289 (7)	0.0258 (7)	−0.0037 (5)	−0.0018 (5)	−0.0063 (6)
O8	0.0186 (6)	0.0261 (7)	0.0245 (7)	−0.0079 (5)	−0.0030 (5)	−0.0043 (6)
N4	0.0197 (8)	0.0170 (8)	0.0179 (7)	−0.0054 (6)	−0.0051 (6)	−0.0023 (6)
N5	0.0157 (7)	0.0179 (8)	0.0245 (8)	−0.0002 (6)	−0.0042 (6)	−0.0022 (6)
N6	0.0168 (8)	0.0185 (8)	0.0365 (9)	−0.0050 (6)	−0.0032 (7)	0.0021 (7)
C16	0.0182 (9)	0.0206 (9)	0.0157 (8)	−0.0052 (7)	−0.0021 (7)	−0.0027 (7)
C17	0.0196 (9)	0.0249 (10)	0.0139 (8)	−0.0088 (7)	−0.0003 (7)	−0.0019 (7)
C18	0.0228 (9)	0.0205 (9)	0.0140 (8)	−0.0073 (7)	−0.0004 (7)	−0.0031 (7)
C19	0.0256 (10)	0.0252 (10)	0.0192 (9)	−0.0121 (8)	0.0015 (7)	−0.0049 (7)
C20	0.0345 (11)	0.0198 (10)	0.0243 (10)	−0.0126 (8)	0.0030 (8)	−0.0058 (8)
C21	0.0297 (10)	0.0196 (10)	0.0218 (9)	−0.0046 (8)	0.0026 (8)	−0.0056 (7)
C22	0.0225 (9)	0.0206 (10)	0.0188 (9)	−0.0055 (7)	0.0019 (7)	−0.0034 (7)
C23	0.0228 (9)	0.0179 (9)	0.0139 (8)	−0.0076 (7)	−0.0015 (7)	−0.0015 (7)
C24	0.0281 (10)	0.0251 (10)	0.0183 (9)	−0.0062 (8)	−0.0067 (7)	−0.0031 (7)
C25	0.0195 (9)	0.0253 (10)	0.0137 (8)	−0.0043 (7)	0.0004 (7)	−0.0034 (7)
C26	0.0154 (8)	0.0204 (9)	0.0190 (9)	−0.0015 (7)	0.0015 (7)	−0.0001 (7)
C27	0.0214 (10)	0.0198 (10)	0.0591 (14)	−0.0076 (8)	−0.0027 (9)	0.0023 (9)
C28	0.0195 (10)	0.0210 (11)	0.0543 (14)	−0.0003 (8)	0.0019 (9)	0.0087 (9)
C29	0.0178 (9)	0.0333 (12)	0.0338 (11)	0.0011 (8)	−0.0035 (8)	0.0069 (9)
C30	0.0217 (9)	0.0272 (11)	0.0255 (10)	−0.0006 (8)	−0.0045 (8)	−0.0020 (8)
O9	0.0207 (9)	0.0158 (8)	0.0298 (9)	−0.0042 (6)	−0.0090 (7)	−0.0047 (6)
O10	0.0165 (11)	0.0150 (14)	0.0404 (16)	−0.0010 (10)	−0.0108 (10)	−0.0061 (12)
O11	0.0210 (9)	0.0140 (9)	0.0291 (9)	−0.0034 (7)	−0.0057 (7)	−0.0047 (7)
O12	0.0183 (14)	0.0172 (14)	0.0385 (11)	−0.0057 (10)	−0.0092 (11)	−0.0085 (12)
C31	0.0151 (10)	0.0154 (11)	0.0150 (10)	−0.0035 (8)	−0.0011 (8)	−0.0010 (8)
C32	0.0178 (14)	0.0120 (11)	0.0167 (12)	−0.0052 (11)	−0.0006 (12)	−0.0022 (9)
C33	0.0155 (12)	0.0170 (13)	0.0212 (12)	0.0009 (10)	−0.0034 (9)	−0.0014 (9)
C34	0.0144 (12)	0.0208 (14)	0.0200 (11)	−0.0009 (11)	−0.0030 (9)	−0.0035 (12)
C35	0.0168 (11)	0.0168 (11)	0.0188 (10)	−0.0049 (9)	0.0001 (8)	−0.0034 (9)
C36	0.0169 (14)	0.0128 (13)	0.019 (2)	−0.0031 (10)	−0.0016 (10)	−0.0014 (10)
C37	0.0178 (12)	0.0158 (13)	0.0164 (11)	−0.0036 (11)	−0.0018 (9)	−0.0013 (9)
O9B	0.017 (3)	0.017 (3)	0.029 (4)	−0.004 (3)	−0.012 (3)	−0.002 (3)
O10B	0.021 (6)	0.013 (5)	0.031 (4)	−0.006 (4)	−0.006 (4)	−0.005 (4)
O11B	0.012 (3)	0.017 (4)	0.030 (4)	0.001 (3)	−0.015 (3)	−0.002 (3)
O12B	0.018 (5)	0.015 (6)	0.039 (6)	−0.004 (4)	−0.014 (4)	−0.002 (5)
C31B	0.014 (3)	0.013 (3)	0.015 (3)	−0.001 (2)	−0.002 (2)	−0.005 (2)
C32B	0.011 (4)	0.008 (3)	0.016 (3)	−0.003 (3)	−0.006 (4)	−0.004 (3)
C33B	0.019 (4)	0.013 (4)	0.024 (4)	−0.006 (4)	0.002 (3)	−0.003 (4)
C34B	0.017 (4)	0.017 (4)	0.024 (3)	−0.003 (3)	0.000 (3)	−0.003 (3)
C35B	0.013 (3)	0.021 (4)	0.021 (3)	−0.004 (3)	−0.006 (3)	0.000 (3)
C36B	0.012 (4)	0.018 (4)	0.017 (4)	0.000 (4)	−0.003 (3)	−0.001 (4)
C37B	0.019 (4)	0.014 (4)	0.018 (4)	0.002 (4)	0.001 (3)	−0.005 (4)

### Geometric parameters (Å, º)

**Table:**

S1—O2	1.4333 (12)	C18—C19	1.397 (3)
S1—O1	1.4361 (12)	C18—C23	1.404 (3)
S1—N1	1.6299 (14)	C19—C20	1.383 (3)
S1—C8	1.7646 (17)	C19—H19A	0.9500
O3—C10	1.241 (2)	C20—C21	1.389 (3)
O4—C2	1.285 (2)	C20—H20A	0.9500
N1—C1	1.449 (2)	C21—C22	1.387 (3)
N1—C9	1.477 (2)	C21—H21A	0.9500
N2—C11	1.363 (2)	C22—C23	1.384 (3)
N2—C10	1.392 (2)	C22—H22A	0.9500
N2—H2	0.88 (2)	C24—H24A	0.9800
N3—C11	1.344 (2)	C24—H24B	0.9800
N3—C12	1.348 (2)	C24—H24C	0.9800
N3—H3	0.78 (2)	C26—C30	1.396 (3)
C1—C2	1.395 (2)	C27—C28	1.374 (3)
C1—C10	1.437 (2)	C27—H27A	0.9500
C2—C3	1.499 (2)	C28—C29	1.377 (3)
C3—C4	1.396 (2)	C28—H28A	0.9500
C3—C8	1.405 (2)	C29—C30	1.375 (3)
C4—C5	1.388 (2)	C29—H29A	0.9500
C4—H4A	0.9500	C30—H30A	0.9500
C5—C6	1.385 (3)	O9—C37	1.238 (3)
C5—H5A	0.9500	O10—C37	1.318 (4)
C6—C7	1.388 (3)	O10—H10	0.91 (3)
C6—H6A	0.9500	O11—C32	1.356 (3)
C7—C8	1.388 (2)	O11—H11	0.81 (3)
C7—H7A	0.9500	O12—C35	1.370 (4)
C9—H9A	0.9800	O12—H12	0.81 (3)
C9—H9B	0.9800	C31—C36	1.400 (4)
C9—H9C	0.9800	C31—C32	1.407 (3)
C11—C15	1.399 (2)	C31—C37	1.471 (3)
C12—C13	1.362 (3)	C32—C33	1.395 (4)
C12—H12A	0.9500	C33—C34	1.386 (4)
C13—C14	1.397 (2)	C33—H33A	0.9500
C13—H13A	0.9500	C34—C35	1.396 (3)
C14—C15	1.377 (2)	C34—H34A	0.9500
C14—H14A	0.9500	C35—C36	1.383 (5)
C15—H15A	0.9500	C36—H36A	0.9500
S2—O6	1.4293 (13)	O9B—C37B	1.245 (8)
S2—O5	1.4295 (13)	O10B—C37B	1.323 (9)
S2—N4	1.6428 (15)	O10B—H10B	0.8400
S2—C23	1.7624 (18)	O11B—C32B	1.356 (8)
O7—C25	1.238 (2)	O11B—H11B	0.8400
O8—C17	1.337 (2)	O12B—C35B	1.373 (9)
O8—H8	0.88 (3)	O12B—H12B	0.8400
N4—C16	1.441 (2)	C31B—C36B	1.394 (9)
N4—C24	1.482 (2)	C31B—C32B	1.400 (8)
N5—C25	1.369 (2)	C31B—C37B	1.464 (8)
N5—C26	1.401 (2)	C32B—C33B	1.394 (9)
N5—H5	0.84 (2)	C33B—C34B	1.388 (9)
N6—C26	1.334 (2)	C33B—H33B	0.9500
N6—C27	1.343 (3)	C34B—C35B	1.393 (8)
C16—C17	1.360 (3)	C34B—H34B	0.9500
C16—C25	1.457 (3)	C35B—C36B	1.378 (10)
C17—C18	1.471 (3)	C36B—H36B	0.9500
			
O2—S1—O1	117.92 (7)	C20—C19—H19A	120.1
O2—S1—N1	108.03 (7)	C18—C19—H19A	120.1
O1—S1—N1	108.36 (7)	C19—C20—C21	121.01 (18)
O2—S1—C8	110.48 (7)	C19—C20—H20A	119.5
O1—S1—C8	109.35 (8)	C21—C20—H20A	119.5
N1—S1—C8	101.39 (8)	C22—C21—C20	120.24 (18)
C1—N1—C9	115.16 (13)	C22—C21—H21A	119.9
C1—N1—S1	114.32 (11)	C20—C21—H21A	119.9
C9—N1—S1	116.52 (11)	C23—C22—C21	118.57 (17)
C11—N2—C10	125.83 (15)	C23—C22—H22A	120.7
C11—N2—H2	121.7 (14)	C21—C22—H22A	120.7
C10—N2—H2	112.4 (14)	C22—C23—C18	122.17 (17)
C11—N3—C12	123.43 (16)	C22—C23—S2	120.38 (14)
C11—N3—H3	117.4 (16)	C18—C23—S2	117.37 (14)
C12—N3—H3	119.1 (16)	N4—C24—H24A	109.5
C2—C1—C10	125.28 (15)	N4—C24—H24B	109.5
C2—C1—N1	121.38 (15)	H24A—C24—H24B	109.5
C10—C1—N1	113.28 (14)	N4—C24—H24C	109.5
O4—C2—C1	123.40 (15)	H24A—C24—H24C	109.5
O4—C2—C3	118.03 (14)	H24B—C24—H24C	109.5
C1—C2—C3	118.55 (15)	O7—C25—N5	123.18 (17)
C4—C3—C8	117.53 (15)	O7—C25—C16	120.74 (17)
C4—C3—C2	120.01 (15)	N5—C25—C16	116.05 (16)
C8—C3—C2	122.46 (15)	N6—C26—C30	122.23 (17)
C5—C4—C3	120.54 (16)	N6—C26—N5	114.40 (16)
C5—C4—H4A	119.7	C30—C26—N5	123.35 (17)
C3—C4—H4A	119.7	N6—C27—C28	123.5 (2)
C6—C5—C4	120.85 (16)	N6—C27—H27A	118.2
C6—C5—H5A	119.6	C28—C27—H27A	118.2
C4—C5—H5A	119.6	C27—C28—C29	117.72 (19)
C5—C6—C7	120.00 (16)	C27—C28—H28A	121.1
C5—C6—H6A	120.0	C29—C28—H28A	121.1
C7—C6—H6A	120.0	C30—C29—C28	120.25 (19)
C8—C7—C6	118.87 (16)	C30—C29—H29A	119.9
C8—C7—H7A	120.6	C28—C29—H29A	119.9
C6—C7—H7A	120.6	C29—C30—C26	118.22 (19)
C7—C8—C3	122.22 (15)	C29—C30—H30A	120.9
C7—C8—S1	120.18 (13)	C26—C30—H30A	120.9
C3—C8—S1	117.59 (13)	C37—O10—H10	107.2 (19)
N1—C9—H9A	109.5	C32—O11—H11	106 (2)
N1—C9—H9B	109.5	C35—O12—H12	108 (3)
H9A—C9—H9B	109.5	C36—C31—C32	119.5 (2)
N1—C9—H9C	109.5	C36—C31—C37	120.9 (2)
H9A—C9—H9C	109.5	C32—C31—C37	119.6 (2)
H9B—C9—H9C	109.5	O11—C32—C33	117.8 (2)
O3—C10—N2	120.69 (15)	O11—C32—C31	123.0 (2)
O3—C10—C1	123.59 (15)	C33—C32—C31	119.2 (2)
N2—C10—C1	115.71 (14)	C34—C33—C32	120.6 (2)
N3—C11—N2	119.99 (15)	C34—C33—H33A	119.7
N3—C11—C15	117.96 (15)	C32—C33—H33A	119.7
N2—C11—C15	122.04 (15)	C33—C34—C35	120.4 (2)
N3—C12—C13	120.29 (16)	C33—C34—H34A	119.8
N3—C12—H12A	119.9	C35—C34—H34A	119.8
C13—C12—H12A	119.9	O12—C35—C36	118.2 (2)
C12—C13—C14	118.25 (16)	O12—C35—C34	122.4 (2)
C12—C13—H13A	120.9	C36—C35—C34	119.4 (2)
C14—C13—H13A	120.9	C35—C36—C31	120.9 (2)
C15—C14—C13	120.70 (16)	C35—C36—H36A	119.6
C15—C14—H14A	119.7	C31—C36—H36A	119.6
C13—C14—H14A	119.7	O9—C37—O10	121.5 (2)
C14—C15—C11	119.34 (15)	O9—C37—C31	122.7 (2)
C14—C15—H15A	120.3	O10—C37—C31	115.8 (2)
C11—C15—H15A	120.3	C37B—O10B—H10B	109.5
O6—S2—O5	119.45 (8)	C32B—O11B—H11B	109.5
O6—S2—N4	107.35 (8)	C35B—O12B—H12B	109.5
O5—S2—N4	108.47 (8)	C36B—C31B—C32B	120.0 (7)
O6—S2—C23	108.59 (8)	C36B—C31B—C37B	120.9 (7)
O5—S2—C23	109.83 (8)	C32B—C31B—C37B	119.1 (6)
N4—S2—C23	101.66 (8)	O11B—C32B—C33B	116.5 (7)
C17—O8—H8	106.5 (16)	O11B—C32B—C31B	124.8 (7)
C16—N4—C24	113.57 (14)	C33B—C32B—C31B	118.6 (7)
C16—N4—S2	112.35 (11)	C34B—C33B—C32B	121.3 (8)
C24—N4—S2	116.08 (12)	C34B—C33B—H33B	119.4
C25—N5—C26	126.35 (16)	C32B—C33B—H33B	119.4
C25—N5—H5	119.4 (16)	C33B—C34B—C35B	119.4 (8)
C26—N5—H5	114.2 (16)	C33B—C34B—H34B	120.3
C26—N6—C27	118.03 (17)	C35B—C34B—H34B	120.3
C17—C16—N4	120.42 (16)	O12B—C35B—C36B	117.0 (9)
C17—C16—C25	121.07 (16)	O12B—C35B—C34B	122.9 (9)
N4—C16—C25	118.42 (15)	C36B—C35B—C34B	120.1 (7)
O8—C17—C16	123.01 (17)	C35B—C36B—C31B	120.6 (9)
O8—C17—C18	114.49 (16)	C35B—C36B—H36B	119.7
C16—C17—C18	122.43 (16)	C31B—C36B—H36B	119.7
C19—C18—C23	118.14 (17)	O9B—C37B—O10B	122.6 (8)
C19—C18—C17	121.45 (16)	O9B—C37B—C31B	122.2 (7)
C23—C18—C17	120.31 (16)	O10B—C37B—C31B	115.2 (8)
C20—C19—C18	119.86 (18)		
			
O2—S1—N1—C1	−62.23 (13)	C16—C17—C18—C23	17.4 (3)
O1—S1—N1—C1	168.95 (11)	C23—C18—C19—C20	−1.0 (3)
C8—S1—N1—C1	53.93 (13)	C17—C18—C19—C20	−177.36 (16)
O2—S1—N1—C9	159.44 (12)	C18—C19—C20—C21	0.7 (3)
O1—S1—N1—C9	30.63 (14)	C19—C20—C21—C22	−0.2 (3)
C8—S1—N1—C9	−84.39 (13)	C20—C21—C22—C23	0.1 (3)
C9—N1—C1—C2	93.30 (19)	C21—C22—C23—C18	−0.4 (3)
S1—N1—C1—C2	−45.60 (19)	C21—C22—C23—S2	176.15 (13)
C9—N1—C1—C10	−84.02 (18)	C19—C18—C23—C22	0.9 (3)
S1—N1—C1—C10	137.08 (13)	C17—C18—C23—C22	177.31 (16)
C10—C1—C2—O4	1.0 (3)	C19—C18—C23—S2	−175.77 (13)
N1—C1—C2—O4	−176.02 (15)	C17—C18—C23—S2	0.6 (2)
C10—C1—C2—C3	−177.70 (15)	O6—S2—C23—C22	−97.44 (15)
N1—C1—C2—C3	5.3 (2)	O5—S2—C23—C22	34.84 (17)
O4—C2—C3—C4	19.5 (2)	N4—S2—C23—C22	149.56 (14)
C1—C2—C3—C4	−161.71 (16)	O6—S2—C23—C18	79.31 (15)
O4—C2—C3—C8	−160.13 (15)	O5—S2—C23—C18	−148.41 (13)
C1—C2—C3—C8	18.6 (2)	N4—S2—C23—C18	−33.69 (15)
C8—C3—C4—C5	−0.6 (2)	C26—N5—C25—O7	3.8 (3)
C2—C3—C4—C5	179.71 (16)	C26—N5—C25—C16	−174.46 (16)
C3—C4—C5—C6	0.4 (3)	C17—C16—C25—O7	−7.8 (3)
C4—C5—C6—C7	−0.1 (3)	N4—C16—C25—O7	175.54 (15)
C5—C6—C7—C8	0.0 (3)	C17—C16—C25—N5	170.47 (16)
C6—C7—C8—C3	−0.2 (3)	N4—C16—C25—N5	−6.2 (2)
C6—C7—C8—S1	−178.95 (14)	C27—N6—C26—C30	−0.9 (3)
C4—C3—C8—C7	0.5 (2)	C27—N6—C26—N5	−178.99 (17)
C2—C3—C8—C7	−179.81 (15)	C25—N5—C26—N6	−164.95 (16)
C4—C3—C8—S1	179.26 (13)	C25—N5—C26—C30	17.0 (3)
C2—C3—C8—S1	−1.1 (2)	C26—N6—C27—C28	1.2 (3)
O2—S1—C8—C7	−99.16 (15)	N6—C27—C28—C29	−0.5 (3)
O1—S1—C8—C7	32.21 (16)	C27—C28—C29—C30	−0.6 (3)
N1—S1—C8—C7	146.49 (14)	C28—C29—C30—C26	0.8 (3)
O2—S1—C8—C3	82.06 (14)	N6—C26—C30—C29	−0.1 (3)
O1—S1—C8—C3	−146.57 (13)	N5—C26—C30—C29	177.80 (17)
N1—S1—C8—C3	−32.29 (15)	C36—C31—C32—O11	−179.4 (3)
C11—N2—C10—O3	4.3 (3)	C37—C31—C32—O11	−0.8 (4)
C11—N2—C10—C1	−174.59 (15)	C36—C31—C32—C33	−1.4 (4)
C2—C1—C10—O3	176.72 (16)	C37—C31—C32—C33	177.3 (2)
N1—C1—C10—O3	−6.1 (2)	O11—C32—C33—C34	179.3 (2)
C2—C1—C10—N2	−4.4 (2)	C31—C32—C33—C34	1.2 (4)
N1—C1—C10—N2	172.79 (14)	C32—C33—C34—C35	−0.1 (4)
C12—N3—C11—N2	178.15 (16)	C33—C34—C35—O12	179.6 (3)
C12—N3—C11—C15	−1.3 (2)	C33—C34—C35—C36	−0.8 (4)
C10—N2—C11—N3	−2.9 (3)	O12—C35—C36—C31	−179.8 (4)
C10—N2—C11—C15	176.52 (15)	C34—C35—C36—C31	0.6 (6)
C11—N3—C12—C13	1.7 (3)	C32—C31—C36—C35	0.5 (6)
N3—C12—C13—C14	−0.6 (3)	C37—C31—C36—C35	−178.1 (3)
C12—C13—C14—C15	−0.8 (3)	C36—C31—C37—O9	−174.1 (3)
C13—C14—C15—C11	1.2 (3)	C32—C31—C37—O9	7.3 (3)
N3—C11—C15—C14	−0.2 (2)	C36—C31—C37—O10	6.5 (4)
N2—C11—C15—C14	−179.62 (15)	C32—C31—C37—O10	−172.1 (3)
O6—S2—N4—C16	−59.05 (13)	C36B—C31B—C32B—O11B	176.7 (16)
O5—S2—N4—C16	170.60 (12)	C37B—C31B—C32B—O11B	−3.3 (16)
C23—S2—N4—C16	54.87 (13)	C36B—C31B—C32B—C33B	−2 (2)
O6—S2—N4—C24	167.90 (12)	C37B—C31B—C32B—C33B	177.7 (10)
O5—S2—N4—C24	37.55 (15)	O11B—C32B—C33B—C34B	−177.8 (9)
C23—S2—N4—C24	−78.18 (14)	C31B—C32B—C33B—C34B	1.3 (17)
C24—N4—C16—C17	87.5 (2)	C32B—C33B—C34B—C35B	0.1 (16)
S2—N4—C16—C17	−46.7 (2)	C33B—C34B—C35B—O12B	177.7 (12)
C24—N4—C16—C25	−95.81 (18)	C33B—C34B—C35B—C36B	0 (2)
S2—N4—C16—C25	129.92 (14)	O12B—C35B—C36B—C31B	−178.8 (17)
N4—C16—C17—O8	−176.38 (15)	C34B—C35B—C36B—C31B	−1 (3)
C25—C16—C17—O8	7.1 (3)	C32B—C31B—C36B—C35B	2 (3)
N4—C16—C17—C18	6.9 (3)	C37B—C31B—C36B—C35B	−178.1 (16)
C25—C16—C17—C18	−169.68 (16)	C36B—C31B—C37B—O9B	−178.4 (16)
O8—C17—C18—C19	16.6 (2)	C32B—C31B—C37B—O9B	1.5 (15)
C16—C17—C18—C19	−166.38 (17)	C36B—C31B—C37B—O10B	1 (2)
O8—C17—C18—C23	−159.65 (16)	C32B—C31B—C37B—O10B	−178.7 (11)

### Hydrogen-bond geometry (Å, º)

**Table:**

*D*—H···*A*	*D*—H	H···*A*	*D*···*A*	*D*—H···*A*
N2—H2···O4	0.88 (2)	1.85 (2)	2.6169 (18)	145.2 (19)
N3—H3···O3	0.78 (2)	1.99 (2)	2.6027 (19)	135 (2)
N3—H3···O3^i^	0.78 (2)	2.19 (2)	2.8034 (19)	136 (2)
O8—H8···O7	0.88 (3)	1.79 (3)	2.5722 (19)	148 (2)
N5—H5···O9	0.84 (2)	2.25 (2)	3.074 (2)	167 (2)
O10—H10···N6	0.91 (3)	1.74 (3)	2.637 (3)	167 (3)
O11—H11···O9	0.81 (3)	1.89 (3)	2.614 (2)	148 (3)
O12—H12···O4^ii^	0.81 (3)	1.94 (3)	2.738 (3)	164 (4)
O10*B*—H10*B*···O4^ii^	0.84	1.67	2.508 (12)	172
O11*B*—H11*B*···O2^i^	0.84	2.57	3.061 (6)	118
O11*B*—H11*B*···O9*B*	0.84	1.89	2.614 (9)	143
O12*B*—H12*B*···N6	0.84	2.10	2.856 (12)	149

Symmetry codes: (i) −*x*+1, −*y*+1, −*z*+1; (ii) *x*−1, *y*, *z*.
